# Long‐Term Incidence of Peri‐Implant Conditions: 25‐Year Results of The Bernese Prospective Cohort Study

**DOI:** 10.1111/cid.70171

**Published:** 2026-07-09

**Authors:** Emilio Couso‐Queiruga, Andrea Roccuzzo, Clemens Raabe, Vivianne Chappuis, Daniel Buser, Manrique Fonseca, Giovanni E. Salvi

**Affiliations:** ^1^ Department of Oral Surgery and Stomatology University of Bern School of Dental Medicine Bern Switzerland; ^2^ Shanghai Perio‐Implant Innovation Center, Institute of Integrated Oral, Craniofacial and Sensory Research, Shanghai Ninth People's Hospital, Shanghai Jiao Tong University School of Medicine, College of Stomatology Shanghai Jiao Tong University, National Center of Stomatology, National Clinical Research Center for Oral Diseases, Shanghai Key Laboratory of Stomatology, Shanghai Research Institute of Stomatology Shanghai China; ^3^ Department of Periodontology, School of Dental Medicine University of Bern Bern Switzerland; ^4^ Department of Reconstructive Dentistry and Gerodontology University of Bern School of Dental Medicine Bern Switzerland

**Keywords:** dental implants, peri‐implantitis, phenotype, titanium

## Abstract

**Objective:**

To assess 25‐year implant survival, success rates, peri‐implant conditions, and variables influencing peri‐implant mucositis (PM), peri‐implantitis (PI), and loss of osseointegration.

**Methods:**

Partially edentulous patients rehabilitated with tissue‐level implant‐supported prostheses were evaluated. Calibrated examiners performed clinical and radiographic assessments, and potential variables for PM and PI were analyzed at the final follow‐up.

**Results:**

At the 10‐ and 25‐year follow‐ups, 303 and 159 patients with 511 and 252 implants, respectively, were included. Implant failure (IF) was 1.2% at 10 years and 6.0% at 25 years, with implant survival rates of 98.8% and 94.0%, respectively (*p* < 0.001). Implant success rates ranged from 97.0% at 10 years to 90.8% at 25 years (*p* < 0.001). IF due to loss of osseointegration increased from 0.6% to 3.6% at the implant level and from 1.0% to 2.5% at the patient level, and were located only in premolar or molar sites. At implant level, peri‐implant health (PH) was 14.4% at 10 and 25 years (*p* = 1.00), while PM decreased from 82.1% to 76.5% (*p* = 0.08), and PI increased from 3.5% to 9.1% (*p* = 0.001). At patient level, PH remained stable between 10 and 25 years, ranging from 10.0% to 10.3% (*p* = 0.94), while PM decreased from 80.0% to 71.6% (*p* = 0.06), and PI increased from 10.0% to 18.1% (*p* = 0.01). In the multivariate analysis, history of periodontitis (OR = 3.87) and facial mucosal thickness (OR = 0.42) were associated with PM, whereas facial mucosal thickness was associated with PI (OR = 0.02).

**Conclusions:**

After 25 years, implant survival and success exceeded 90%. Loss of osseointegration accounted for approximately 60% of IF. While PI and IF increased over time, PM decreased but remained prevalent.

## 1 Introduction

Over the past five decades, implant dentistry has evolved from an experimental approach into a highly predictable and widely adopted modality for the rehabilitation of partially and fully edentulous patients. In this context, implant‐supported prostheses (ISPs) have enabled a wide spectrum of clinical scenarios, ranging from simple to highly complex rehabilitations, demonstrating long‐term survival rates exceeding 95% [1, 2, 3, 4]. As a result, the global use of ISPs has expanded substantially, with the number of ISPs placed worldwide projected to grow at an annual rate of approximately 8% through 2030 [5]. This sustained growth is primarily driven by population aging, the high prevalence of dental‐related conditions (i.e., caries lesions, periodontitis) [6, 7, 8], and the high patient satisfaction and positive impact on quality of life of this tooth replacement option [9, 10, 11, 12, 13]. Nevertheless, despite the widespread success of ISPs, peri‐implantitis (PI) has emerged as a major concern due to its high prevalence, the challenges in achieving predictable treatment outcomes to restore and maintain peri‐implant health (PH), and its socioeconomic impact [14, 15, 16, 17, 18, 19].

According to the diagnostic criteria established at the 2017 World Workshop on the Classification of Periodontal and Peri‐Implant Diseases and Conditions, peri‐implant mucositis (PM) is defined as inflammation confined to the peri‐implant mucosa, presenting with bleeding on probing (BOP) and/or suppuration (SUP), but without progressive bone loss beyond initial remodeling [20]. In contrast, PI is characterized by these same inflammatory signs combined with radiographic evidence of progressive bone loss [21, 22]. Based on these criteria, a recent meta‐analysis incorporating 20 studies reported a weighted mean prevalence of PM of 63.0% at the patient level, and 59.2% at the implant level, while PI affected 25.0% of patients and 18.0% of implants [23]. Consequently, as the number of implants placed worldwide continues to rise, a corresponding increase in the global burden of PM and PI is expected.

Several long‐term studies have reported implant‐related outcomes with follow‐up periods of 20 years or longer [24, 25, 26, 27, 28, 29, 30]. However, due to heterogeneity among studies, long‐term evidence on titanium implants remains scarce, especially with respect to implant failure (IF), implant success, and peri‐implant conditions in selected clinical scenarios within specialized academic settings. Therefore, the primary aim of this long‐term cohort study was to assess IF and success rates, as well as peri‐implant conditions, after 25 years of follow‐up. A secondary objective was to analyze the influence of relevant variables on PM, PI, and loss of osseointegration.

## 2 Materials and Methods

### 2.1 Experimental Design, Center, and Ethical Approval

This long‐term cohort study was conducted at the Department of Oral Surgery and Stomatology at the University of Bern School of Dental Medicine (Switzerland) in accordance with the principles of the 2018 Declaration of Helsinki. The study protocol was approved by the Ethics Committee for Clinical Studies of the State of Bern, Switzerland (KEK‐BE‐No. 2023‐02279) and was registered in ClinicalTrials.gov (NCT06599320). The first comprehensive clinical and radiographic evaluation was performed between January 2010 and September 2011, and the second between September 2024 and May 2025. This report adheres to the Reporting of Observational Studies in Epidemiology (STROBE) guidelines [31].

### 2.2 Eligibility Criteria and Recruitment

All health records of adult subjects who underwent tooth replacement therapy with ISPs between 1997 and January 2001 and were included in previously reported studies [1, 32] were reviewed for potential inclusion. Patients expressing interest in participating were initially pre‐screened via telephone. At a subsequent in‐person visit, patients were provided with detailed information regarding the study objectives, timeline, and clinical and radiographic evaluations. Before enrollment, all participants reviewed and signed an informed consent form that described the study design, potential benefits, and possible risks. Inclusion criteria at time of implant placement were: (1) age ≥ 18 years; (2) partially edentulous; (3) presence of at least one TL titanium implant with a sandblasted and acid‐etched (SLA) and including a hybrid surface design; and (4) restoration with a cement‐ or screw‐retained ISP. Exclusion criteria for the 25‐year follow‐up included: (1) refusal to undergo further comprehensive examinations and (2) presence of disabilities or conditions impairing the comprehension, reading, or signing of the informed consent.

### 2.3 Clinical Data Collection

During the follow‐up visit, full comprehensive oral evaluations were performed by two calibrated periodontists (E.C‐Q. and A.R.). Calibration involved a protocol review meeting and a preliminary joint assessment of five randomly selected patients to ensure consistency and standardization across assessment methods. Clinical measurements were obtained using a UNC‐15 periodontal probe (Hu‐Friedy, Chicago, USA) and included PD, BOP, and SUP at six sites per tooth and implant: mesio‐facial, mid‐facial, disto‐facial, mesio‐lingual, mid‐lingual, and disto‐lingual. The presence of plaque was recorded at the same six sites. Keratinized mucosa width (KMW) was measured on the mid‐facial aspect. Facial mucosal thickness (FMT) was directly measured at implant sites using a standard no. 20 endodontic finger spreader (Kerr, Kloten, Switzerland) at 3 mm apical to the mucosal margin, perpendicular to the long axis of the ISP, as previously described [33, 34]. Patient demographics, medical and dental histories, history of periodontitis, recorded as a binary variable, based on documented periodontal treatment or radiographic evidence of bone loss at non‐adjacent teeth, surgical and prosthetic variables, participation in a supportive peri‐implant care program, implant location, and other relevant factors were collected using a structured questionnaire.

### 2.4 Radiographic Data Collection

A digital periapical radiograph (Xios XG Supreme, Size 2; Dentsply Sirona, Charlotte, USA) centered on the region of interest was obtained using the parallel technique with standardized stock film holders (XCP, Dentsply Sirona). Additionally, a panoramic radiograph was acquired for diagnostic evaluation. To ensure high‐quality data and consistency, two independent and calibrated examiners (E.C‐Q. and C.R.) evaluated the radiographs from the 10‐ and 25‐year follow‐up evaluations using an open‐source software (ImageJ, U.S. NIH, Bethesda, MD, USA). The calibration session was performed using the first 15 consecutive images. For each radiograph, the known thread pitch distances of each implant were used to convert pixel measurements into millimeters. First, a horizontal line corresponding to the implant platform was drawn, serving as a reference for perpendicular vertical lines (parallel to the implant's long axis) to the first radiographically visible bone‐to‐implant contact at the mesial and distal sites (DIB) [1, 35, 36]. In cases where individual measurements differed by more than 0.2 mm, the two authors verified final data accuracy and consistency by reaching a consensus on the exact position of the distance to the implant shoulder and repeating the measurements to ensure precision. The mean of the measurements from both examiners, as well as the mesial and distal values, served as the final measurement values. Intra‐ and inter‐rater agreement exceeded 0.90.

### 2.5 Definitions

**FIGURE 1:**
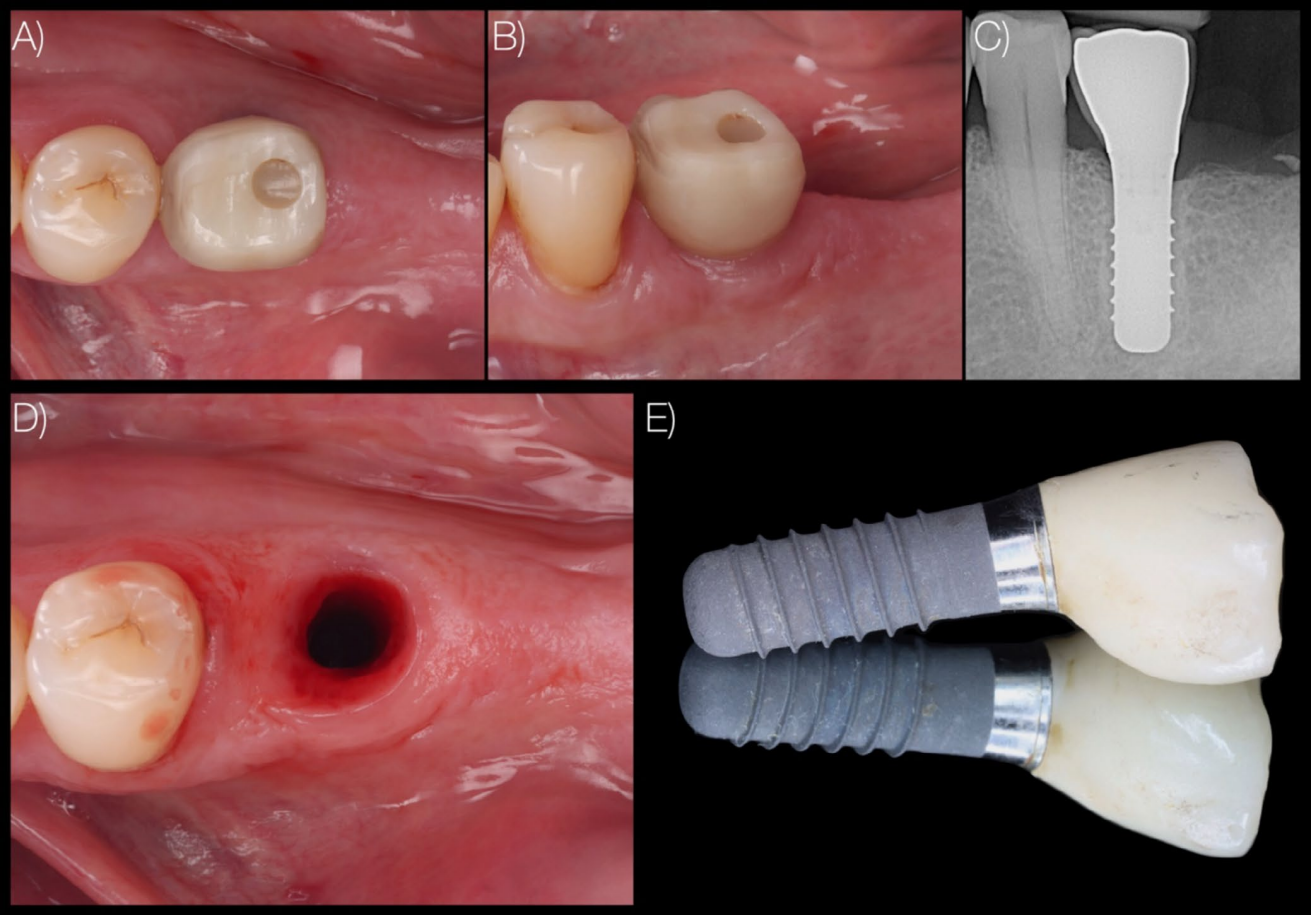
Clinical and radiographic images illustrating a case of loss of osseointegration. Panels (A) and (B) show the occlusal and buccal aspects of the implant, respectively, with clinical mobility and without signs of inflammatory peri‐implantitis. Panel (C) demonstrates peri‐implant radiographic features suggestive of sheathing and corticalization surrounding the implant. Panel (D) presents the occlusal view of the site following implant explanation, and Panel (E) depicts the retrieved implant.

IF was defined as the loss of an implant identified during the clinical examination, and the underlying reasons (e.g., peri‐implantitis) were retrieved from the clinical records. Loss of osseointegration was recorded as a specific reason for IF. Loss of osseointegration was defined as loosening of the implant fixture, evidenced by mobility in the absence of clinical or radiographic signs of peri‐implantitis, and not attributable to any history of accidental trauma, as shown in Figure 1.

Implant success criteria were described as previously reported [37] and have been utilized in several long‐term studies with dental implants at the University of Bern [1, 38, 39, 40]. An implant was considered successful when it demonstrated:

- Absence of persistent subjective complaints such as pain, foreign body sensation, and/or dysesthesia
- Absence of peri‐implant infection with suppuration.
- Absence of mobility.
- Absence of a continuous radiolucency around the implant.

Based on the European Federation of Periodontology S3‐level clinical practice guideline on the prevention and treatment of peri‐implant diseases [41], and the 2017 World Workshop Classification in Periodontal and Peri‐implant Diseases and Conditions [21], the following definitions were applied:

- *PH:* defined as the absence of clinical signs of inflammation, ≤ 1 spot with BOP, and no SUP, absence of bone loss beyond crestal bone level changes resulting from initial bone remodeling after delivery of the definitive ISP.
- *PM:* defined as the presence of > 1 spot with BOP, or presence of a line of bleeding or profuse bleeding at any location and/or SUP on gentle probing. Absence of bone loss beyond the crestal bone level changes resulting from initial bone remodeling after delivery of the definitive ISP.
- *PI:* defined as the presence of BOP and/or SUP, increased probing depths (PD), and the presence of bone loss beyond crestal bone level changes resulting from initial bone remodeling.

### 2.6 Statistical Analyzes

All analyzes were performed using the statistical software R, version 4.4.2, with the packages “tidyverse,” “robustlmm,” “multpois,” “geepack,” “DHARMa,” “gtsummary,” and “DescTools.”

Patient‐ and implant‐related characteristics, as well as clinical and radiographic outcomes, were descriptively summarized by reporting means and standard deviations for continuous variables and frequencies and percentages for categorical variables.

Differences between the 10‐year and 25‐year follow‐ups on the implant level were tested using robust linear mixed regression models for continuous outcomes (standard mixed models showed deviations from normality and were influenced by outliers) and multinomial‐Poisson GLMM for nominal response data (categorical outcomes). In both cases, either a random intercept for the patient or a random slope for the follow‐up year with a random intercept for the patient (if it increased goodness‐of‐fit) was modeled to account for repeated measurements at several time points and multiple implants within a patient. Goodness‐of‐fit of models was assessed by examining model residuals. Crestal bone levels were calculated by subtracting the transmucosal machined collar height from the recorded measurements.

GEE logistic regression models, with an exchangeable covariance structure (i.e., assuming implants to be exchangeable within a patient), were used to assess the association between implant‐related characteristics and the odds of PM and PI compared to PH at the 25‐year follow‐up. Thus, each model compared implants affected by the condition of interest to implants classified as PH at the 25‐year examination. Only univariable models were run for PI and loss of osseointegration due to the limited number of cases. For PM, variables of interest were first screened using univariable models and were then included in a final multivariable model if the *p*‐value was ≤ 0.10. The final multivariable model included three estimators, with a ratio of min (events, non‐events) to estimators of 27/3 = 9, allowing adequate generalizability. Goodness‐of‐fit of logistic models was assessed using the le Cessie–van Houwelingen–Copas–Hosmer test. The multivariable model was also checked for collinearity. Thus, each model compared implants affected by the condition of interest to implants classified as PH at the 25‐year examination.

FMT was adjusted for the presence of inflammation by subtracting 0 mm for PH, 0.5 mm for PM, and 1.0 mm for PI, as previously reported [42, 43, 44]. The corrected FMT was then compared across health status groups using generalized GEE logistic regression models while accounting for the respective explanatory variables (all ratios above 6.8). The analysis of potential explanatory variables was performed using data collected at the 25‐year examination. Finally, all *p*‐values equal to or less than 0.05 were considered statistically significant.

## 3 Results

### 3.1 Patient Sample Characteristics

Out of 303 patients who had received 511 implants and had been followed for a mean of 10.6 ± 0.7 years, 159 patients (86 women [54.1%] and 73 men [45.9%]) with a mean age of 70 ± 13 years and a total of 252 implants were available for assessment after a mean follow‐up of 25.1 ± 0.9 years. This corresponds to a patient‐level dropout rate of 47.5% and an implant‐level dropout rate of 50.7% between the 10 and 25‐year follow‐up. Reasons for dropouts during follow‐up are presented in Table 1.

**TABLE 1:** Reasons for participation attrition during the follow‐up period.

Reasons for participation attrition	10 years follow‐up *N* = 55	25 years follow‐up *N* = 144	Total *N* = 199
Death	6 (11%)	22 (15%)	28 (14%)
Illness	9 (16%)	9 (6%)	18 (9%)
Distance	16 (29%)	24 (17%)	40 (20%)
Could not be reached	6 (11%)	54 (38%)	60 (30%)
Not interested	17 (31%)	35 (24%)	52 (26%)
Pregnancy	1 (2%)	0 (0%)	1 (0.5%)

Among the 159 patients, 9 patients had IF. Therefore, a total of 150 subjects were included in the clinical and radiographic analyzes at the 25‐year follow‐up visit. Six patients were heavy smokers, eight were light smokers, and two had a history of smoking, having ceased smoking at least 1 year ago. Fifteen patients had well‐controlled diabetes (HbA1c < 7%), and history of periodontitis was observed in 38 patients. Regarding oral hygiene, 125 patients reported brushing at least twice daily and performing interdental cleaning at least once per day, while 18 and 28 patients, respectively, reported brushing once per day and no use of interdental cleaning. With respect to supportive peri‐implant care, only four patients did not attend any supportive peri‐implant care visits, while the remaining patients attended at least one annual visit. One patient with one ISP presented with recurrence after a previous history of peri‐implantitis.

Details of implant site characteristics at both follow‐up time points are presented in Table 2, while Table 3 summarizes the clinical and radiographic outcomes. The majority of implants evaluated at 25 years supported cement‐retained (81.3%) reconstructions, the mean FMT was 2.47 ± 0.66 mm, and only 7% of implant sites showed plaque accumulation. At baseline, the primary reasons for tooth loss were caries lesions (*n* = 130), endodontic‐related problems (*n* = 68), trauma (*n* = 63), periodontitis (*n* = 62), agenesia (*n* = 45), and fractures (*n* = 39).

**TABLE 2:** Summary of implant, site, and bone augmentation characteristics at different time points.

	10 years follow‐up *N* = 505	25 years follow‐up *N* = 237	*p*
Diameter			0.76
3.3 mm	16 (3.1%)	8 (3.2%)	
4.1 mm	279 (54.6%)	128 (50.8%)	
4.8 mm	216 (42.3%)	116 (46.0%)	
Implant length			0.97
≤ 10 mm	334 (65.4%)	164 (65.1%)	
12 + mm	177 (34.6%)	88 (34.9%)	
Implant platform			0.99
3.5 mm	16 (3.2%)	8 (3.2%)	
4.8 mm	422 (82.6%)	203 (80.6%)	
6.5 mm	73 (14.3%)	41 (16.3%)	
Location			0.42
Mandible	275 (53.8%)	147 (58.3%)	
Maxilla	236 (46.2%)	105 (41.7%)	
			0.92
Incisors	54 (10.6%)	21 (8.3%)	
Canines	39 (7.6%)	18 (7.1%)	
Premolars	219 (42.9%)	103 (40.9%)	
Molars	199 (38.9%)	110 (43.7%)	
Bone augmentation procedures			0.96
None	358 (70.1%)	181 (71.8%)	
Simultaneous	86 (16.8%)	41 (16.3%)	
Staged	67 (13.1%)	30 (11.9%)	
			0.95
Guided bone regeneration	90 (17.6%)	41 (16.3%)	
Maxillary sinus floor augmentation	63 (12.3%)	30 (12.3%)	

**TABLE 3:** Mean and standard deviation of clinical and radiographic outcomes at different time points.

Clinical and radiographic outcomes	10 years follow‐up *N* = 505	25 years follow‐up *N* = 237	*p*
Mean probing depth (mm)	3.27 ± 0.80	3.02 ± 1.12	0.0004*
Mean bleeding on probing	0.57 ± 0.33	0.42 ± 0.23	< 0.0001*
Suppuration on probing			0.006*
No	503 (99.6%)	229 (96.6%)	
Yes	2 (0.4%)	8 (3.4%)	
Keratinized mucosal width (mm)	2.49 ± 1.20	2.17 ± 1.37	0.0002*
Mean crestal bone levels (mm)	0.01 ± 0.96	−0.26 ± 1.19	< 0.0001*
DIB Mesial (mm)	2.51 ± 0.89	2.89 ± 1.25	< 0.0001*
DIB Distal (mm)	2.59 ± 0.87	2.97 ± 1.17	< 0.0001*
DIB Total (mm)	2.55 ± 0.88	2.93 ± 1.21	< 0.0001*

Abbreviations: DIB: first radiographically visible bone‐to‐implant contact; mm: millimeters.

* Statistical significance.

### 3.2 Assessment of Attrition Bias: Re‐Evaluated Participants Versus Participants Lost to Follow‐Up

When comparing patients lost to follow‐up between the 10‐year and 25‐year assessments with those examined at the 25‐year follow‐up, no statistically significant differences were observed in age at surgery, sex, implant status, baseline characteristics (Table 2), or clinical outcomes (Table 3). However, trends toward higher mean BOP (0.62 vs. 0.54 sites; *p* = 0.09) and PI (0.61 vs. 0.52 sites; p = 0.09) were observed among patients lost to follow‐up.

### 3.3 Implant Survival and Success Rates, and Peri‐Implant Conditions

During the 10‐year evaluation, the IF rate was 1.2%, of which 0.6% (*n* = 3 implants) was attributed to loss of osseointegration and the remaining 0.6% (*n* = 3 implants) to PI. At 25 years, a total of six implants (2.4%) were lost due to PI and nine implants (3.6%) due to loss of osseointegration, resulting in a cumulative IF rate of 6.0% over the 25‐year follow‐up. This represents an absolute increase of 4.8% in IF between the two time points (*p* < 0.001). Accordingly, implant survival rates at 10 and 25 years were 98.8% and 94.0%, respectively. No implant fractures were observed over the 25 years. Implant success rates at 10 and 25 years were 97.0% and 90.8%, respectively (*p* < 0.001).

**FIGURE 2:**
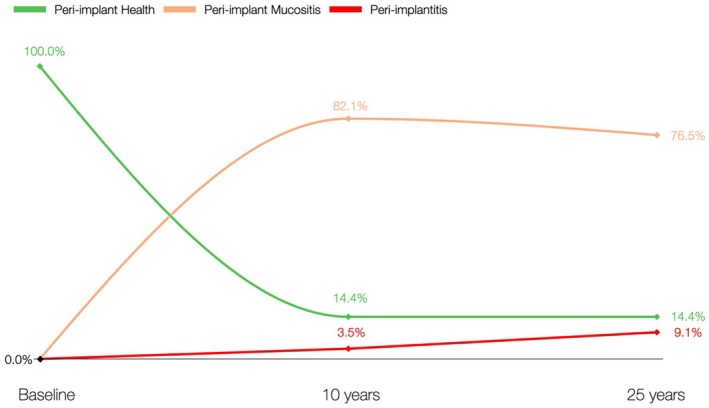
Implant‐level peri‐implant health, peri‐implant mucositis, and peri‐implantitis across the follow‐up interval.

At the 10‐year follow‐up, 73 sites (14.4%) exhibited PH, 417 (82.1%) PM, and 18 (3.5%) PI. At 25 years, 35 sites (14.4%) presented PH (*p* = 1.00), 186 (76.5%) PM (*p* = 0.08), and 22 (9.1%) PI (*p* = 0.001). Figure 2 shows the peri‐implant conditions over time at the implant level.

**FIGURE 3:**
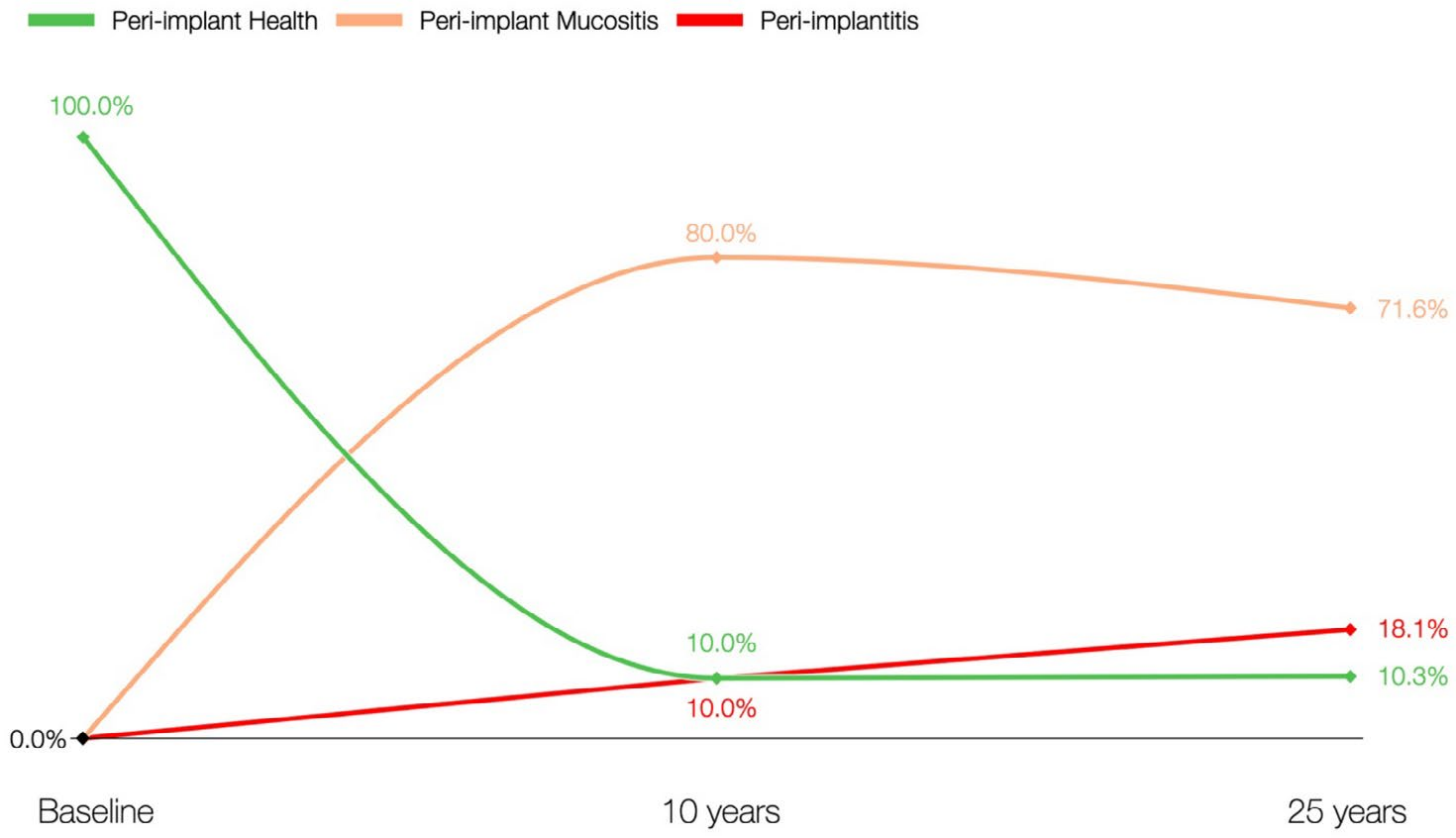
Patient‐level peri‐implant health, peri‐implant mucositis, and peri‐implantitis across the follow‐up interval.

**FIGURE 4:**
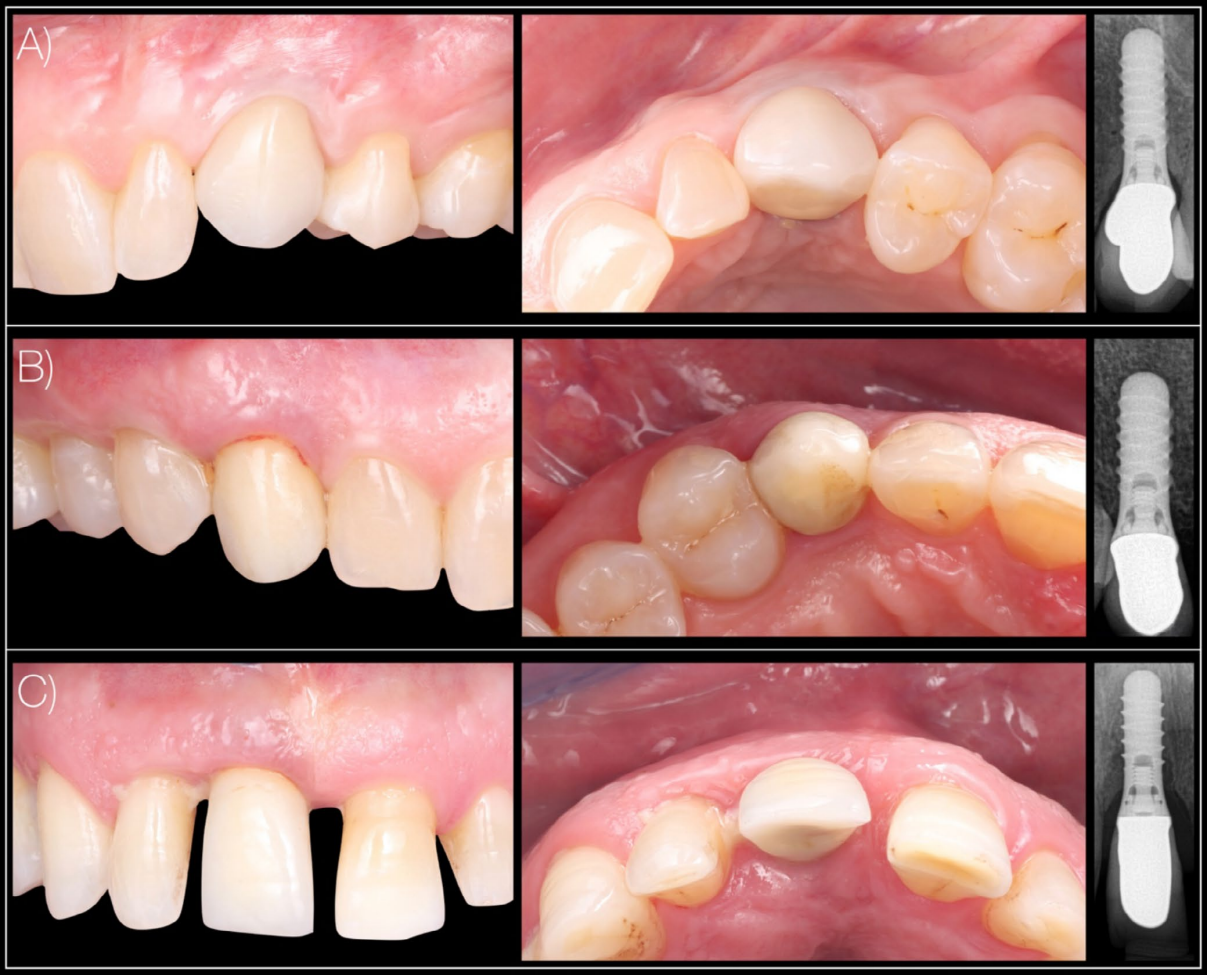
Clinical and radiographic images exemplifying cases of peri‐implant health (A), peri‐implant mucositis (B), and peri‐implantitis (C) at the 25‐year follow‐up. (A) Case restored by Professor Urs Belser, Chairman Emeritus, Division of Fixed Prosthodontics & Occlusion, School of Dental Medicine, University of Geneva, Geneva, Switzerland.

At the patient level, 30 individuals (10.0%) were diagnosed with PH, 240 (80.0%) with PM, and 30 (10.0%) with PI at 10 years. At 25 years, 16 patients (10.3%) had PH (*p* = 0.94), 111 (71.6%) PM (*p* = 0.06), and 28 (18.1%) PI (*p* = 0.01). Figure 3 depicts peri‐implant conditions over time at the patient level. Examples of cases diagnosed with PH, PM, and PI at the 25‐year follow‐up are displayed in Figure 4. The clinical and radiographic characteristics of the peri‐implant conditions are presented in Table 4.

**TABLE 4:** Clinical and radiographic characteristics of the peri‐implant conditions at 25 years.*

Clinical and radiographic characteristics	Peri‐implant health	Peri‐implant mucositis	Peri‐implantitis	*p*
Mean crestal bone levels (mm)	−0.04 ± 0.72	0.08 ± 0.94	3.09 ± 1.02	< 0.001*
Keratinized mucosal width (mm)	2.37 ± 1.29	2.08 ± 1.33	2.81 ± 1.87	0.11
Mean probing depth (mm)	2.64 ± 0.90	2.97 ± 1.06	4.43 ± 1.33	0.001*
Mean bleeding on probing	0.08 ± 0.08	0.47 ± 0.18	0.60 ± 0.32	< 0.001*
Mean plaque	0.09 ± 0.25	0.07 ± 0.15	0.09 ± 0.26	0.40
Suppuration on probing				0.002*
No	35 (100.0%)	182 (97.8%)	12 (75.0%)	
Yes	0 (0.0%)	4 (2.2%)	4 (25.0%)	

* Statistical significance.

### 3.4 Factors Associated With Peri‐Implant Mucositis

Univariable analysis comparing PH and PM identified two factors with *p*‐values ≤ 0.05: history of periodontitis and FMT. In the multivariable analysis, both factors remained significantly associated with PM. An OR of 0.42 indicates that each additional millimeter of FMT was associated with a 58% lower odds of PM (*p* = 0.05). History of periodontitis (OR = 3.87, *p* = 0.05) increased the odds of this condition, meaning that these patients were almost four times more likely to present with PM than those without such a history. Univariable and multivariable analyzes are summarized in Tables 5 and 6.

**TABLE 5:** Univariable analysis of possible factors associated with peri‐implant mucositis.

Factors	Odds ratio (95%‐CI)	*p*
Gender		0.31
Male	Reference	
Female	1.54 (0.67, 3.56)	
Age	1.01 (0.98, 1.04)	0.67
Implant diameter		0.85
3.3 mm	Reference	
4.1 mm	1.14 (0.17, 7.49)	
4.8 mm	0.97 (1.44, 6.54)	
Implant length		0.63
≤ 10 mm	Reference	
12 + mm	0.85 (0.45, 1.61)	
Implant platform		0.90
3.5 mm	Reference	
4.8 mm	1.02 (1.53, 6.74)	
6.5 mm	1.31 (0.16, 10.8)	
Location		0.41
Mandible	Reference	
Maxilla	0.70 (0.29, 1.67)	
Position		0.39
Incisors	Reference	
Canines	0.50 (0.09, 2.70)	
Premolars	0.73 (0.18, 2.95)	
Molars	1.19 (0.28, 4.95)	
Bone augmentation procedures		0.93
None	Reference	
Guided bone regeneration	0.88 (0.31, 2.51)	
Maxillary sinus floor augmentation	1.18 (0.30, 4.58)	
Bone grafting		0.38
None	Reference	
Simultaneous	0.65 (0.26, 1.65)	
Staged	2.22 (0.34, 14.7)	
Prosthesis retention		0.80
Cement retained	Reference	
Screw retained	1.13 (0.44, 2.96)	
Facial mucosal thickness	0.36 (0.19, 0.67)	0.001*
History of Periodontitis		0.04*
No	Reference	
Yes	3.94 (1.08, 14.3)	

*Note:* Effect estimates are presented as odds ratios with 95% confidence intervals in parentheses.

* Statistical significance.

**TABLE 6:** Multivariable analysis of explanatory variables associated with peri‐implant mucositis.

Explanatory variables	Odds ratios	*p*
Facial mucosal thickness	0.42 (0.22, 0.79)	0.05*
History of periodontitis	3.87 (1.00, 15.0)	0.05*

*Note:* Effect estimates are presented as odds ratios with 95% confidence intervals in parentheses.

* Statistical significance.

### 3.5 Factors Associated With Peri‐Implantitis

Univariable regression models comparing PH and PI showed that FMT (*p* < 0.0001) was significantly associated with PI. History of periodontitis also demonstrated a strong trend toward significance (*p* = 0.07). An OR of 0.02 indicates that each additional millimeter of FMT was associated with a 98% lower odds of PI. Finally, history of periodontitis led to higher odds for PI (OR = 5.12, *p* **=** 0.07). However, these individuals were more than five times more likely to present with PI than those without such a history. Factors associated with PI are shown in Table 7.

**TABLE 7:** Univariable analysis of possible factors associated with peri‐implantitis.

Factors	Odds ratio (95% CI)	*p*
Gender		1.00
Male	Reference	
Female	1.00 (0.30, 3.27)	
Age	0.99 (0.94, 1.04)	0.56
Implant diameter		0.93
3.3 mm	Reference	
4.1 mm	1.07 (0.33, 3.48)	
4.8 mm	0.96 (0.24, 3.80)	
Implant length		0.97
≤ 10 mm	Reference	
12 + mm	0.99 (0.55, 1.79)	
Implant platform		0.50
3.5 mm	Reference	
4.8 mm	0.96 (0.24, 3.80)	
6.5 mm	2.33 (0.32, 17.0)	
Location		0.72
Mandible	Reference	
Maxilla	1.20 (0.44, 3.31)	
Position		0.52
Incisors	Reference	
Canines	0.37 (0.05, 2.53)	
Premolars	0.51 (0.11, 2.30)	
Molars	1.59 (0.16, 15.5)	
Bone augmentation procedures		0.64
None	Reference	
Guided bone regeneration	0.51 (0.13, 2.05)	
Maxillary sinus floor augmentation	63 (12.3%)	
Bone grafting		0.51
None	Reference	
Simultaneous	0.44 (0.11, 1.83)	
Staged	0.63 (0.05, 7.58)	
Prosthesis retention		0.49
Cement retained	Reference	
Screw retained	0.54 (0.09, 3.15)	
Facial mucosal thickness	0.02 (0.00, 0.14)	< 0.0001*
History of periodontitis		0.07
No	Reference	
Yes	5.12 (0.88, 29.8)	

*Note:* Effect estimates are presented as odds ratios with 95% confidence intervals in parentheses.

* Statistical significance.

### 3.6 Factors Associated With Loss of Osseointegration

When patients with loss of osseointegration were compared with those presenting PH at the 25‐year follow‐up, no variables were significantly associated with loss of osseointegration in the univariable analyzes. This was likely attributable to the limited number of loss of osseointegration cases (*n* = 9), resulting in insufficient statistical power. Nevertheless, all implants with loss of osseointegration were located in premolar or molar sites (9/37, 24.3%), whereas no cases of loss of osseointegration were observed in incisor or canine sites (0/7, 0.0%), although this difference did not reach statistical significance (*p* = 0.30). An identical distribution and *p*‐value were observed for prosthesis retention type, as all implants with loss of osseointegration were cement‐retained (9/37, 24.3%), while no loss of osseointegration cases occurred among screw‐retained implants (0/7, 0.0%).

## 4 Discussion

To the best of our knowledge, this cohort study provides the most comprehensive long‐term evidence on commercially available TL titanium implants with a hybrid surface design and their corresponding ISPs, reporting 25‐year implant survival and success rates, peri‐implant conditions, and associated factors for PM and PI.

An IF rate of 1.2% at 10 years and 6.0% at 25 years was observed. This translates into a survival rate of 98.8% at 10 years and 94.0% at 25 years. These outcomes compare favorably with those reported in other long‐term investigations. For example, a meta‐analysis estimated a 10‐year implant‐level survival rate of 96.4%, with a sensitivity analysis indicating a survival of 93.2% [13]. Over longer follow‐up periods, Becker and colleagues observed an 88.03% survival rate for titanium plasma‐sprayed implants after a maximum follow‐up of 23 years [24]. Donati and coworkers also reported a survival rate of 87.8% at 20 years follow‐up [26]. Similarly, Jung and coworkers reported survival rates of 89.3%, 90.2%, and 93.8% at 22–24 years in sites treated with horizontal bone augmentation using resorbable collagen membranes, non‐resorbable ePTFE barriers, or in pristine bone, respectively [45]. In another long‐term investigation, Roccuzzo [46] and collaborators observed an overall 20‐year survival rate of 93.0% [29]. More recently, a meta‐analysis of data extracted from retrospective and prospective studies after 20 years of function revealed mean survival rates of 88.0% and 92.0%, respectively [27]. The higher survival rate observed in the present cohort may be related to differences in study eligibility criteria, implant systems, lower PI rates, as well as variability in supportive peri‐implant care, oral hygiene practices, and patient‐related local, systemic, and behavioral factors.

To our knowledge, this long‐term cohort study is the first to specifically assess loss of osseointegration as a cause for IF in a longitudinal study. We observed 3.6% at the implant level and 2.5% at the patient level after 25 years. Interestingly, all of these cases were observed in premolar and molar sites and occurred in association with cement‐retained ISP. This clinical entity, although infrequent in shorter follow‐up periods, has been extensively reported in the medical field [47, 48, 49, 50, 51]. These findings suggest that loss of osseointegration may represent an underrecognized cause of IF in the context of implant therapy and, therefore, should be considered in future investigations, consensus meetings, and world workshops. This would allow a more precise characterization of its etiology (i.e., occlusal trauma), and generate relevant data regarding its prevalence and potential associations with patient‐, local‐, prosthetic‐, surgical‐, and implant‐related factors [52, 53, 54, 55], as well as with potential management of this condition [56, 57, 58].

Over the observation period, a favorable long‐term success rate above 90% was observed at 25 years. However, an increase in PI was noted at both the implant and patient levels. At the patient level, PI increased from 10.0% at 10 years to 18.1% at 25 years and was higher than that observed at the implant level (3.5% and 9.1% at 10 and 25 years). In contrast, PM showed a slightly lower prevalence, with patient‐level rates decreasing from 80.0% to 71.6% and at the implant level from 82.1% to 76.5% between 10 and 25 years. PH remained largely stable over time, with an implant level rate of 14.4% and patient level rates of 10.0% and 10.3% at 10 and 25 years, respectively. Prevalence for PM is higher, while PI is lower than that reported by Roccuzzo et al. who observed PM rates of 67.9% and 47.6%, and for PI rates of 10.6% and 33.3% after 10 and 20 years, respectively. However, a similar trend was observed in both studies, characterized by an increasing incidence of PI and a concomitant decrease in PM. Their cohort included patients with a history of periodontitis and with different levels of adherence to a tailored supportive peri‐implant care program, which may explain the higher disease burden, in addition to the evaluation of only four peri‐implant sites, and the subtle differences in diagnostic criteria for peri‐implant diseases [30]. Our findings align with those reported in a recent systematic review and meta‐analysis applying the 2017 World Workshop definitions, which reported weighted mean prevalences of PM of 63.0% at the patient level and 59.2% at the implant level, and PI of 25.0% and 18.0%, respectively [23]. Notably, most studies in that review had considerably shorter follow‐up periods, reinforcing the concept that longer time in function may be associated with an increased likelihood of onset and progression of PI, as reflected in this study.

Several factors may explain the relatively low prevalence of PI at 25 years in our cohort despite the long follow‐up. Most patients demonstrated adequate oral hygiene and regularly attended supportive peri‐implant care, the restrictive diagnosis criteria used, survivor bias, and exclusion of implants previously lost. Although PI remained as a biological complication over 25 years follow‐up, its prevalence was lower than that of PM, which remained relatively high. These findings underscore the importance of preventive measures to minimize further PI. The prevalence of smoking was low, and all patients diagnosed with diabetes had adequate glycemic control. In addition, the hybrid surface design implants were placed in optimal three‐dimensional positions, with the microrough surface inserted slightly below the bone crest at the time of surgery, leading to a smooth implant surface at the transcrestal peri‐implant sulcus [1, 59]. Outcomes from long‐term studies have indicated that early bone loss, leading to exposure of the microrough surface, is a predictor of PI [60]. A recent study by Moser and colleagues demonstrated that early bone loss prior to prosthetic loading is associated with a substantially increased risk of IF and PI. Specifically, PI was diagnosed in 63.2% of implants presenting early bone loss, compared with 5.2% of implants without early bone loss after a minimum follow‐up of 5 years. At the patient level, PI was observed in 68.4% of patients with early bone loss versus 6.1% of patients without early bone loss [61]. Accordingly, this hybrid implant design may contribute to reduced rates of IF and PI, as proposed by Tarnow in 1993 [62]. When these factors are not adequately controlled, as in this cohort, previous studies identified them as potential risk factors, indicators, predisposing, or contributing conditions for the onset and progression of PM and PI [63, 64, 65, 66, 67, 68, 69, 70, 71, 72, 73, 74, 75].

The influence of a variety of relevant variables associated with PM and PI was also assessed in this study. The history of periodontitis was strongly associated with more than a fivefold and approximately fourfold higher likelihood of PI and PM, respectively. Our findings align with those from previous studies [30, 67, 76] and recently published systematic reviews [75, 77]. Notably, this association persisted despite the case‐selected nature of the present cohort. Considering the histopathological, microbiological, and immunoinflammatory distinctions between PM and PI and periodontitis, a plausible explanation is that persistent microbial dysbiosis, in combination with local, systemic, surgical, and behavioral factors in susceptible individuals, may precipitate a destructive immunoinflammatory response, thereby promoting the onset and progression of PM and PI [78, 79, 80, 81, 82, 83, 84]. Therefore, comprehensive preventive strategies across all levels of patient care, including primordial, primary, and secondary prevention, are essential to reduce the odds of PM and PI and their associated multifaceted consequences in susceptible subjects, even in patients demonstrating adequate oral hygiene and adherence to peri‐implant supportive care.

Interestingly, FMT was associated with reduced odds of both PM and PI. Sites with a thicker FMT were associated with lower odds of both PM and PI compared to sites with a thinner FMT. A previous study reported a prevalence of PM of 46.3% in sites with thin FMT versus 41.2% in sites with thick FMT, although no significant association was observed with PI [85]. Conversely, two other studies found minimal differences in the prevalence of PI between sites with thin or thick mucosal thickness [69, 86]. Our findings, however, align with a cohort study where the mucosal thickness was associated with lower odds of PM [87], and with a recent cross‐sectional study by our group that evaluated the conditions around bone‐level and TL titanium implants after approximately 11 years of function. In this study, a 1 mm increase in FMT reduced the odds of PM and PI by 56% and 83%, respectively [42]. This may partially explain why peri‐implant mucosal dehiscence is most often treated with soft tissue phenotype modification therapies, which can achieve stable long‐term outcomes even in cases with insufficient hard tissue support [88, 89, 90], or in situations where horizontal bone augmentation may be biologically limited or less predictable due to the patient‐specific phenotypic bone boundaries [91, 92]. Nevertheless, no association between KMW and PM and PI was observed in our cohort. In contrast, other investigators have reported that insufficient peri‐implant KMW (≤ 2 mm) in erratic compliers seems to be associated with peri‐implant diseases [93]. However, our observations are consistent with a recent umbrella systematic review that concluded that current evidence is insufficient to establish a direct causal relationship between KMW and PI [94], but contrary to a recent long‐term study on the same TL included in the present investigation (Andrea Roccuzzo et al.). This raises the question of whether, in individuals with adequate oral hygiene measures, high compliance, and lower risk profiles, a minimum band of KMW is necessary, or if adequate mucosal thickness can compensate for its deficiency and serve as a protective local phenotypic factor against PM and PI. Finally, heterogeneity in assessment methodologies may contribute to discrepancies across studies and potentially lead to misclassification or underestimation of specific components of the peri‐implant phenotype [95, 96, 97], thereby masking their true effect.

Despite adhering to strict methodological standards, this study has several limitations. First, only titanium TL implants from a single manufacturer were included. This limits the external validity of the findings reported to other implant systems with different macro‐ or microscopic features. Nonetheless, the homogeneity of the implants used in this study reduced variability and allowed for a more controlled comparison within a well‐established implant system. Second, all implant placements were carried out by specialists in an academic setting. Therefore, the outcomes may not fully reflect those observed in routine dental practice, particularly in settings with different levels of clinician experience or less standardized surgical, prosthetic, and supportive peri‐implant care protocols. Third, the cohort was drawn exclusively from a Swiss population; consequently, the findings may not be fully generalizable to populations from other countries with differing demographic compositions, behavioral habits, cultural contexts, socio‐economic status, oral health awareness, and health profiles. Fourth, the loss to follow‐up at 25 years, although comparable to rates reported in other long‐term studies, may have introduced attrition and survivor bias, influencing the representativeness and trueness, and potential generalizability of the reported outcomes. Fifth, the definition of peri‐implant conditions (e.g., PD ≥ 6 mm, BOP, or bone loss beyond crestal bone level changes resulting from initial bone remodeling) may have led to misdiagnosis. Similarly, time‐to‐event data were not collected during the follow‐up period. In addition, the analysis of potential explanatory variables (e.g., FMT) was performed with the last follow‐up clinical and radiographic evaluations, precluding causal inference. Therefore, the possibility of reverse causation cannot be excluded. Finally, PI was only assessed on an univariate basis, and for PM, the multivariate model only consisted of parameters with *p*‐values ≤ 0.10 and ≤ 0.05 from univariable screening, potentially excluding relevant confounding and correcting variables. Future studies should aim to validate these results in larger and more diverse cohorts, across different implant systems and clinical settings, using standardized, reproducible, and precise methods for outcome assessment.

## 5 Conclusions

Within the limitations of this long‐term cohort study and considering the potential impact of cohort attrition, the following conclusions may be drawn:

– Long‐term implant survival and success rates were above 90% in partially edentulous patients treated with TL titanium implants characterized by a hybrid surface design.
– Loss of osseointegration was responsible for approximately 60% of IF.
– PI increased over 25 years.
– PM remained high over 25 years.
– A history of periodontitis was significantly associated with PM.
– Greater FMT was associated with a lower prevalence of PM and PI.

## Author Contributions

Emilio Couso‐Queiruga and Giovanni E. Salvi conceived and designed the idea. Emilio Couso‐Queiruga, Andrea Roccuzzo, Clemens Raabe, Manrique Fonseca, and Giovanni E. Salvi contributed to clinical and digital data collection. Emilio Couso‐Queiruga and Clemens Raabe contributed to digital data analysis. Emilio Couso‐Queiruga led the writing. Andrea Roccuzzo, Clemens Raabe, Vivianne Chappuis, Daniel Buser, Manrique Fonseca, and Giovanni E. Salvi contributed to data interpretation and critically revised the manuscript. All authors approved the final version and agreed to be responsible for all aspects of the work.

## Funding

This work was supported by the International Team for Implantology (1574‐2021).

## Ethics Statement

The study received approval from the Standing Ethics Committee for Clinical Studies of the State of Bern, Switzerland (KEK‐BE No. 2023‐02279).

## Conflicts of Interest

The authors declare no conflicts of interest.

## Data Availability

Data are available from the corresponding author upon reasonable request, but are not publicly accessible due to privacy and ethical restrictions.
